# Subarachnoid hemorrhage and cerebral vasospasm – Literature review

**Published:** 2013-06-25

**Authors:** AV Ciurea, C Palade, D Voinescu, DA Nica

**Affiliations:** * Neurosurgery Clinic, „Bagdasar-Arseni” Emergency Hospital; ** Neurosurgical Department, Elias Emergency Hospital, Bucharest; *** Neurosurgical Department, „Sf. Pantelimon” Emergency Hospital, Bucharest

**Keywords:** Apoptosis, Early brain injury, Nitrous oxide, Hypoxia induced factor-1, Nitrous oxide

## Abstract

Subarachnoid hemorrhage represents a serious disease with high mortality and morbidity. Two important areas are becoming the central research interest of subarachnoid hemorrhage: cerebral vasospasm and early brain injury. The authors have reviewed the major contributions in experimental subarachnoid hemorrhage documented in the medical literature in the past 5 years.

Treatments interfering with nitric oxide - or endothelin-pathways continue to show antispasmotic effects in experimental models of subarachnoid hemorrhage. Inflammation and oxidative stress play a vital role in the pathophysiology of cerebral vasospasm.

Apoptosis, a relevant cause of early brain injury after subarachnoid hemorrhage, also underline the etiology of cerebral vasospasm. Future research studies will continue to elucidate the pathophysiological pathways and treatment modalities targeting cerebral vasospasm and early brain injury, enabling an improvement in outcome for patients with subarachnoid hemorrhage.

## Introduction

Aneurismal subarachnoid hemorrhage (SAH) represents a serious disease that carries a high mortality and morbidity [69]. The incidence is of approximately 10/100,000 people per year [**39**]. Approximately 11% of the patients die before receiving medical attention, additional 40% of the patients die within 4 weeks after admission to hospital [**23**]. Up to 30% of the survivors exhibit significant morbidity and will depend on others for activities of daily living [**69**]. Nearly 50% of the survivors develop cognitive dysfunctions in the long time and never return to their previous status [**33**]. Despite the advances in diagnosis and treatment of SAH, effective therapeutic interventions are still limited and clinical outcomes remain disappointing. There is substantial evidence that two main issues contribute to the significant mortality and morbidity associated with SAH: cerebral vasospasm (CVS) and early brain injury (EBI). Cerebral vasospasm occurs usually on day 3 after SAH, peaks at days 6 and 8, and lasts for 2-3 weeks [**70**]. CVS has been considered the major cause of high mortality and poor outcome [**17**], thus researches have been primarily focused on vasospasm and its sequelae during the last several decades. Nevertheless, the success rate with regard to improvement o f outcome is limited [**57**]. In addition, although around 70% of the patients may present angiographic cerebral vasospasm after SAH, only about 30% will exhibit neurological deficits [**17**].

Whether the vasospasm is the only major cause of significant mortality and morbidity associated with SAH is questionable. More recently, early brain injury following SAH has also been linked to mortality and morbidity in SAH patients [**7,21**]. Early brain injury refers to the immediate injury to the brain, within the first 72 h following SAH. The underlying pathophysiological mechanisms include the immediate global ischemic brain injury caused by an acute increase in intracranial cerebral pressure and decrease in cerebral blood flow, initiation of cell death signaling, blood brain barrier breakdown, brain edema and inflammation [**5,8,10,35,51,68**]. In this literature review, we intend to realize an overview of the major advances in experimental SAH, published during the last 10 years, with an emphasis on the major pathophysiological pathways involved in the development of vasospasm and early brain injury, as well as treatment strategies targeting vasospasm and early brain injury.

### Vasospasm and Nitric Oxide Pathway 

 The pathway of endothelial nitric oxide has been implicated as a major pathophysiological mechanism for the development of cerebral vasospasm [**16,53**]. NO, produced by the endothelial nitric oxide synthase (eNOS) in the cerebrovascular endothelium, diffuses to adjacent smooth muscle cells and stimulates soluble guanylyl cyclase (sGC), leading to generation of cGMP. cGMP activates intracellular calcium channels, transporting free Ca2+ into intracellular compartment and relaxing smooth muscle cells [**16**]. Vatter et al. aimed to characterize the endothelium-NO-cGMP-dependent pathway of cerebral arteries altered by delayed cerebral vasospasm, since the efficacy of the treatment targeting vasospasm by interfering with the NO-pathway at different levels seems to be inconsistent [**66**]. The results suggested that the endothelium- NO-cGMP dependent relaxation is morphologically and functionally preserved in the major cerebral arteries during vasospasm in a rat model, by immunohistochemical analysis of eNOS and sGC expression and measuring the vasorelaxant effect of sodium nitroprusside (SNP), acetylcholine and 8-bromo-cGMP on rat BA ring segments.

 Hence, the conclusion that treatment of cerebral vasospasm aiming at the endothelium-NO-cGMP-dependent pathway seems to be practicable [**66**]. Osuka et al. showed that in a rat single-hemorrhage model, eNOS was significantly activated in the basilar arteries at an early stage after the onset of SAH, accompanied by the upregulation of AMP activated protein kinase (AMPK a) [**52**]. The AMPKa-eNOS signaling pathway could be important in modulating cerebral blood flow in mild vasospasm [**52**]. As therapeutic intervention, 17b-estradiol benzoate (E2) was reported to attenuate vasospasm and preserve the eNOS expression by activating estrogen receptor subtype a (ERa) in a rat double-hemorrhage model [**38,62**]. Furthermore, the same laboratory demonstrated that E2 mediated vasoprotection through inhibiting SAH-induced increase in expression levels of inducible nitric oxide synthase (iNOS) via NF-кB signaling pathway [**61**]. 

### Cerebral Vasospasm and Endothelin Pathway

Endothelin plays an important role in the development of cerebral vasospasm after SAH. Endothelin-1, a potent vasoconstrictor, was isolated from cultured porcine endothelial cells by Yanagisawa and colleagues in 1988 [**73**] and it acts by two specific receptors, ET(A) and ET(B) [**55**]. Elevated levels of endothelin have been found in the cerebrospinal fluid of patient after SAH [71]. Activation of ET(A) receptor on the vascular smooth muscle cells results in vasoconstriction, whereas ET(B1) receptor subtype, expressed on the vascular endothelial cells, mediates the vasorelaxant effects of endothelin. ET(B2) receptor subtype is localized on smooth muscle cells and causes vasoconstriction [**76**], but the expression and function of the ET(B) receptor subtypes after SAH is not well known. Vatter et al. demonstrated an unchanged immunohistochemical expression of the ET(B) receptor, which was observed exclusively in the endothelium, during the development of delayed cerebral vasospasm in a rat double-hemorrhage model [**67**]. Furthermore, they showed that sarafotoxin 6c (S6c), an ET (B) receptor agonist, did not cause vasoconstriction under resting tension in basilar artery segments. However, after preconstruction, activation of ET(B) receptor by S6c results in a vasodilatation in sham-operated rat, which decreased time-dependently after SAH [**67**]. Thereby, a functionally relevant ET(B2) receptor-mediated vasoconstriction of the cerebrovasculature during vasospasm seems to be absent [**67**]. In contrast, Ansar et al. reported that SAH induces the upregulation of ET(B) receptor (mRNA and protein levels) in the cerebrovascular smooth muscle cells over the first 48 h in a rat single-hemorrhage model [**1**]. 

The discrepancy between the findings may relate to differences in the SAH models (double- vs. single-hemorrhage) and time point of ET(B) receptor measurement (on day 3 and 5 vs. the first 48 h). In this regard, further investigations are warranted. Schubert et al. determined the role of endothelin in the acute phase (the first minutes to hours) after SAH in a rat single hemorrhage model. Prophylactic treatment with clazosentan, the endothelin receptor antagonist, was shown not to affect peracute cerebral perfusion pressure -dependent hypoperfusion, but will block the continuous cerebral blood flow reduction [**58**].

### Cerebral Vasospasm and Hypoxia Inducible Factor-1 (HIF-1)

 HIF-1 is a transcription factor involved in various biological processes including energy metabolism, angiogenesis, erythropoiesis, cell survival and apoptosis [**14,22,28**]. HIF-1 is a key molecule in the pathophysiological response to hypoxia and oxidative stress and regulates more than 40 genes including vascular endothelial growth factor (VEGF), erythropoietin, BNIP3, and glucose transporter-1 [**59**]. Previous experimental data suggest that HIF-1 may play a dual role by activating both prosurvival and pro death pathways in the central nervous system in the settings of ischemic stroke and cerebral hemorrhage [**3,54**]. Likewise, HIF-1 protein expression was shown to be upregulated on Day 7 after SAH in a rat double-hemorrhage model [**25**]. Administration of deferoxamine (DFO), a HIF-1 activator, on Day 4 increased the HIF-1 protein expression and activity on Day 7 and attenuated the basilar artery vasospasm in the same SAH model [**25**]. However, Yan et al. reported that HIF-1 protein expression and activity was significantly increased at 24 h after SAH in a rat monofilament puncture model. 2-Methxyestradiol (2ME2), a HIF-1 inhibitor, administered at 1 h after SAH attenuated cerebral vasospasm and the neurological deficits [**72**]. 

 It has been speculated that HIF-1 could also play a prosurvival and pro death role in the context of SAH. Activation of HIF-1 at an early stage after SAH may be detrimental whereas HIF-1 stimulation at a later stage could be neuroprotective. However, the HIF-1 downstream cascades mediating these beneficial and detrimental effects after SAH remain to be further elucidated. 

### Vasospasm and Inflammation 

 Substantial evidence implicates a critical role of pro-inflammatory cascades in the development and maintenance of cerebral vasospasm after SAH [**18**]. Administration of simvastatin after onset of SAH was shown to attenuate vasospasm and decrease perivascular granulocyte migration at 72 h after SAH in a single-hemorrhage model, suggesting the efficacy of simvastatin targeting vasospasm may dependent on its anti-inflammatory effects [**46**]. Zhou et al. demonstrated that SAH induces an increase in the NF-kB DNA-binding activity and the mRNA levels of TNFa, IL-1b, intercellular adhesion molecule-1 and vascular cell adhesion molecule-1 on Day 5 after SAH in a double-hemorrhage model [**70**].

 The administration of pyrrolidine dithiocarbamate (PDTC), a NF-кB inhibitor, reversed the aforementioned SAH-induced effects and attenuated the vasospasm after SAH, indicating NF-кB mediated pro-inflammatory response in SAH may contribute to the development of cerebral vasospasm [**75**]. N-benzyl-oxycarbonyl- Val Ala Asp-fluoromethyl ketone (Z-VAD-FMK), a caspase inhibitor, was shown to reduce the cerebral vasospasm on Day 2 after SAH in a rabbit single-hemorrhage model, which is associated with a decrease in the IL-1b release into the cerebrospinal fluid and the levels of caspase-1 and IL-1b in macrophages infiltrating into the subarachnoid space [**27**]. 

 Monocyte chemoattractant protein-1(MCP-1), a potent chemokine attracting macrophage, has been implicated in the detrimental inflammatory processes associated with stroke and other disorders in the central nervous system [**11,34**]. Lu et al. found that the mRNA and protein levels of MCP-1 increased in a parallel time course to the development of cerebral vasospasm (peaked on day 5) in a rat double-hemorrhage model, suggesting that specific MCP-1 antagonists may be beneficial to prevent vasospasm caused by SAH [**41**]. Experimental data from Bowman et al. indicates that inflammatory cytokines, in particular IL-6, are involved in the development of vasospasm in the rat femoral artery model [**6**]. 

### Cerebral Vasospasm / Early Brain Injury and Oxidative Stress

 Superoxide anion levels in the cerebrospinal fluid have been shown to be increased parallel to the development of cerebral vasospasm [**47**]. Treatment strategies inhibiting free radical generating enzymes or scavenging free radical have been reported to attenuate vasospasm in animal models of SAH [**4,26,45**]. Karaoglan et al. demonstrated that resveratrol, a stilbene polyphenol and tyrosine kinase inhibitor, reduced SAH-induced cerebral vasospasm in a rat single-hemorrhage model. This protective effect is associated with decreased lipid peroxidation levels in brain and serum, and increased superoxide dismutase expression compared to the untreated group [**30**]. 

 Hypersensitivity of the basilar artery to hydroxyl radicals has been implicated to underlie the pathogenesis of cerebral vasospasm after SAH [**48**]. Furthermore, free radicals can damage neurons and other major cell types in the brain by enhancing lipid peroxidation, protein oxidation and degradation, and DNA damage, which results in endothelial injury and blood brain barrier (BBB) breakdown by initiating apoptotic cascades or necrosis processes [**2,37,42**]. 

 Inhibition of oxidative stress has been shown to prevent apoptosis and BBB permeability. Recently, Ersahin et al. showed that administration of antioxidant melatonin prevented BBB breakdown, reduced brain edema and neurological deficits in a rat single-hemorrhage model [**20**]. Using transgenic rats, Endo et al. demonstrated that reduction in oxidative stress by superoxide dismutase (SOD) over expression results in decreased apoptosis in an endovascular perforation SAH model [**19**]. In addition, this antiapoptotic effect contributed to the activation of Akt/glycogen synthase kinase-3beta (GSK-3b) signaling pathway [**19**]. Another antioxidant, Mexiletine was shown to attenuate apoptosis of endothelial cells and prevent cerebral vasospasm in a rabbit single hemorrhage model [**60**], suggesting a cross-talk between vasospasm and early brain injury and that the attenuation of vasospasm may be contributed at least partially to the preservation of endothelium integrity due to the reduction in oxidative stress. 

### Cerebral Vasospasm and Apoptosis

Apoptosis is one of the major pathophysiological components of early brain injury, and may also play a significant role in the etiology of cerebral vasospasm in the setting of SAH. Zhou et al. demonstrated previously that treatment with caspase inhibitors reduced endothelial apoptosis and vasospasm in a dog model of experimental SAH [**74**]. 

 Recombinant human erythropoietin (rhEPO) has been shown to attenuate the vasospasm in a rabbit double-hemorrhage model by inhibiting the endothelial apoptosis and these beneficial effects may be mediated by the activation of JAK2/STAT3 signaling pathway [**13**]. Likewise, JAK2 has been shown to be activated in the arterial wall after SAH and inhibition of JAK2 by AG490 aggravated the endothelium apoptosis and cerebral vasospasm by downregulation of bcl-2 and bcl-xL [**12**].

 Reduction in apoptosis by administration of PFT-a, a p53 inhibitor, results in preventing severe cerebral vasospasm and blood brain barrier (BBB) breakdown in a rat monofilament puncture model of SAH by down- regulating caspase 8, cytochrome C, apoptosis inducing factor (AIF) and caspase 3 in the basilar arteries [**9**]. The mentioned study results and previously experimental data implicate that prevention of apoptosis might attenuate cerebral vasospasm after SAH [**8,12,13,56**].

 One of the possible mechanisms could be the reduced function of endothelial cells in SAH to prevent smooth muscle cell proliferation and vasoconstriction by generating inhibiting factors such as endothelial NO synthase or absence of endothelial vasodilative ET(B) receptor-mediated vasorelaxation due to the endothelial cell injury. Further studies are warranted to explore the cross talk between early brain injury and cerebral vasospasm after SAH. 

### Statin and Cerebral Vasospasm 

Previous experimental and clinical studies have suggested that treatment with 3-hydroxy-3-methylglutaryl coenzyme A (HMGCoA) reductase inhibitors, also referred to as statins, promotes endothelial function, and reduces cerebral vasospasm in the setting of SAH [**36,43,44,49**]. Recently, Sugawara et al. demonstrated that the administration of simvastatin attenuated cerebral vasospasm and improved neurological outcomes in a rat’s SAH endovascular perforation model. Moreover, this simvastatin-mediated neuroprotection depends on the activation of PI3K/Akt/eNOS pathway [**63**]. Prophylactic treatment with atorvastatin has been shown to reduce apopto apoptotic cell death, prevent blood brain barrier disrupt, attenuate cerebral vasospasm and improve neurological outcome in a rat perforating SAH model. 

 Down regulation of caspase 3 and caspase 8 may contribute to the atorvastatin-induced neuroprotection in the context of SAH [**15**]. 

### Thrombin and Cerebral Vasospasm 

 Thrombin, a serine protease coagulation protein, has been previously implicated in the pathogenesis of cerebral vasospasm after SAH and blood brain barrier permeability in animal models of ischemic stroke and intracerebral hemorrhage [**32,50**]. Thrombin activity in patient cerebrospinal fluid has been shown to coincide with the development of cerebral vasospasm and the degree of SAH [**31**]. Inhibition of thrombin activity by antithrombin III attenuated cerebral vasospasm, which associated with decreased immunoactivity of MAPK in smooth muscle cells of basilar artery [**65**]. Recently, Kai et al. Demonstrated that inhibition of proteinase activated receptor 1 (PAR1), which mediates thrombin’s vascular effects [**24**], by E5555 blocked the upregulation of PAR1 expression and the hypercontractile response of the basilar artery to thrombin in a rabbit double-SAH model [**29**]. Moreover, the administration of argatroban, a direct thrombin inhibitor, has been shown to prevent early brain injury after SAH in a rat intravascular perforation model, including reduction in cell death, brain edema and expression of inflammatory marker, and preservation of BBB integrity [**64**]. 

 These new data further provide evidence that inhibition of thrombin may exhibit powerful neuroprotection in the setting of SAH by targeting both cerebral vasospasm and early brain injury, the two major events of SAH, which contribute to the significant mortality and morbidity of SAH. 

## Conclusions

Research efforts with regard to SAH during the last 10 years have been continued focusing on exploring the pathogenesis of cerebral vasospasm and more recently, that of early brain injury. Endothelium- NO-cGMP dependent vasodilatation appears to be conserved in the major cerebral arteries during cerebral vasospasm and treatment strategies interfering this pathway seem to be practicable. 

 Endothelin appears to play a significant role not only in the development of delayed cerebral vasospasm, but also in the acute vasoconstriction after SAH. Prophylactic endothelin antagonism has been shown to prevent the acute hypoperfusion after SAH. The role of endothelin B receptor subtype after SAH remains to be clarified. 

 HIF-1 seems to play a dual role in the setting of SAH. Inhibition of HIF-1 at early stage and activation of HIF-1 at later stage have been shown to prevent vasospasm after SAH. Further researches to evaluate the involved HIF-1 downstream cascades are warranted. 

 The inflammatory response as well as oxidative stress associated to SAH may play biphasic roles in the pathophysiology after SAH, as it has been suggested for ischemic stroke [**40**]. Pro-inflammatory reactions and free radicals at acute stage of SAH may contribute to cell death and cerebral vasospasm, whereas they might be required for neurovascular remodeling and neurogenesis in later stage. In this regard, further studies are needed to elucidate the time course and evaluate the optimal time windows for treatment targeting the inflammatory reactions and oxidative stress after SAH.

 Early brain injury and cerebral vasospasm are the two major components contributing to brain injury after SAH. Cell death after SAH plays an important role not only in the long-term morbidity of SAH, but also possibly in the etiology of cerebral vasospasm. 

 Cerebral vasospasm in turn results in hypoperfusion of the related brain areas and may also trigger cell death processes. Molecular pathways underlying early brain injury after SAH remains further to be elucidated. Given the complexity of the pathogenesis in SAH, therapeutic modalities interfering with different pathophysiological pathways of SAH, and interventions targeting both cerebral vasospasm and early brain injury appear to be more desirable.

## References

[1] Ansar  S, Vikman  P (2007). Cerebrovascular ETB, 5 HT1B, and AT1 receptor upregulation correlates with reduction in regional CBF after subarachnoid hemorrhage. Am J Physiol Heart Circ Physiol.

[2] Ayer  RE, Zhang  JH (2008). Oxidative stress in subarachnoid haemorrhage: significance in acute brain injury and vasospasm. Acta Neurochir Suppl.

[3] Baranova  O, Miranda  LF (2007). Neuron specific inactivation of the hypoxia inducible factor 1 alpha increases brain injury in a mouse model of transient focal cerebral ischemia. J Neurosci.

[4] Barbosa  MD, Arthur  AS (2001). The novel 5 lipoxygenase inhibitor ABT 761 attenuates cerebral vasospasm in a rabbit model of subarachnoid hemorrhage. Neurosurgery.

[5] Bederson  JB, Germano  IM (1995). Cortical blood flow and cerebral perfusion pressure in a new noncraniotomy model of subarachnoid hemorrhage in the rat. Stroke.

[6] Bowman  G, Bonneau  RH (2006). A novel inhibitor of inflammatory production (CNI 1493) reduces rodent post hemorrhagic vasospasm. Neurocrit Care.

[7] Broderick  JP, Brott  TJ (1994). Initial and recurrent bleeding are the major causes of death following subarachnoid hemorrhage. Stroke.

[8] Cahill  J, Zhang  JH (2009). Subarachnoid hemorrhage: is it time for a new direction?. Stroke.

[9] Cahill  J, Calvert  JW (2006). Vasospasm and p53 induced apoptosis in an experimental model of subarachnoid hemorrhage. Stroke.

[10] Cahill  WJ, Calvert  JH (2006). Mechanisms of early brain injury after subarachnoid hemorrhage. J Cereb Blood Flow Metab.

[11] Che  X, Ye  W (2001). Monocyte chemoat tractant protein 1 expressed in neurons and astrocytes during focal ischemia in mice. Brain Res.

[12] Chen  G, Wu  J (2008). Potential role of JAK2 in cerebral vasospasm after experimental subarachnoid hemorrhage. Brain Res.

[13] Chen G, Zhang  S (2009). Effects of recombinant human erythropoietin (rhEPO) on the JAK2/STAT3 pathway and endothelial apoptosis in the rabbit basilar artery after subarachnoid hemorrhage. Cytokine.

[14] Chen W, Ostrowski  RP (2009). Prodeath or pro survival: two facets of hypoxia inducible factor 1 in perinatal brain injury. Exp Neurol.

[15] Cheng  G, Wei  L (2009). Atorvastatin ameliorates cerebral vasospasm and early brain injury after subarachnoid hemorrhage and inhibits caspase dependent apoptosis pathway. BMC Neurosci.

[16] Dietrich  HH, Dacey  RG (2000). Molecular keys to the problems of cerebral vasospasm.. Neurosurgery.

[17] Dorsch  NW (1995). Cerebral arterial spasm a clinical review. Br J Neurosurg.

[18] Dumont  AS, Dumont  RJ (2003). Cerebral vasospasm after subarachnoid hemorrhage: putative role of inflammation. Neurosurgery.

[19] Endo  H, Nito  C (2007). Reduction in oxidative stress by superoxide dismutase overexpression attenuates acute brain injury after subarachnoid hemorrhage via activation of Akt/glycogen synthase kinase 3beta survival signaling. J Cereb Blood Flow Metab.

[20] Ersahin  M, Toklu  HZ (2009). Melatonin reduces experimental subarachnoid hemorrhage induced oxidative brain damage and neurological symptoms. J Pineal Res.

[21] Frykholm  P, Andersson  JL (2004). Haemodynamic and metabolic disturbances in the acute stage of subarachnoid haemorrhage demonstrated by PET. Acta Neural Scand.

[22] Helton  R, Cui  J (2005). Brain specific knock out of hypoxia inducible factor 1 alpha reduces rather than increases hypoxic ischemic damage. J Neurosci.

[23] Hijdra  A, Braakman  R (1987). Aneurysmal subarachnoid hemorrhage. Complications and outcome in a hospital population. Stroke.

[24] Hirano  K, Kanaide  H (2003). Role of proteinase activated receptors in the vascular system. J Atheroscler Thromb.

[25] Hishikawa  T, Ono  S (2008). Effects of deferoxamine activated hypoxia inducible factor 1 on the brainstem after subarachnoid hemorrhage in rats. Neurosurgery.

[26] Horky  LL, Pluta  RM (1998). Role of ferrous iron chelator 2,2’ dipyridyl in preventing delayed vasospasm in a primate model of subarachnoid hemorrhage. J Neurosurg.

[27] Iseda  K, Ono  S (2007). Antivasospastic and antiinflammatory effects of caspase inhibitor in experimental subarachnoid hemorrhage. Neurosurg.

[28] Jiang  Y, Wu  J (2002). Hypoxia inducible factor 1a accumulation in the brain after experimental intracerebral hemorrhage. J Cereb Blood Flow Metab.

[29] Kai  Y, Hirano  K (2007). Prevention of the hypercontractile to thrombin by proteinase activated receptor 1 antagonist in subarachnoid hemorrhage. Stroke.

[30] Karaoglan  A, Akdemir  O (2008). The effect of resveratrol on vasospasm after experimental subarachnoid hemorrhage in rats. Surg Neurol.

[31] Kasuya  H, Shimizu  T (1998). Thrombin activity in CSF after SAH is correlated with the degree of SAH the persistence of subarachnoid clot and the development vasospasm. . Acta Neurochir (Wien).

[32] Kitaoka  T, Hua  Y (2002). Delayed argatroban treatment reduces edema in a rat model of intracerebral hemorrhage. Stroke.

[33] Kreiter  KT, Copeland  D (2002). Predictors of cognitive dysfunction after subarachnoid hemorrhage. Stroke.

[34] Kumagai N, Chiba Y (2007). Involvement of pro inflammatory cytokines and microglia in an age associated neurodegeneration model, the SAMP10 mouse. Brain Res.

[35] Kusaka  G, Ishikawa  M (2004). Signaling pathways for early brain injury after subarachnoid hemorrhage. J Cereb Blood Flow Metab.

[36] Laufs  U, Liao  JK (1998). Post transcriptional regulation of endothelial nitric oxide synthase mRNA stability by Rho GTPase. J BioChem.

[37] ewen  A, Matz  P (2000). Free radical pathways in CNS injury. J Neurotrauma.

[38] Lin  CL, Shih  HC (2006). The effect of 17beta estradiol in attenuating experimental subarach noid hemorrhage induced cerebral vasospasm. J Neurosurg.

[39] Linn  FH, Rinkel  GJ (1996). Incidence of subarachnoid hemorrhage: role of region, year, and rate of computed to mography: a meta analysis. Stroke.

[40] Lo  EH (2008). A new penumbra: transitioning from injury into repair after stroke. Nat Med.

[41] Lu  H, Shi  JX (2009). Expression of monocyte chemoattractant protein 1 inthe cerebral artery after experimental subarachnoid hemmorrhage. Brain Res.

[42] Matz  PG, Copin  JC (2000). Cell death after exposure to sub arachnoid hemolysate correlates inversely with expression of CuZn superoxide dismutase. Stroke.

[43] McGirt MJ, Blessing  R (2006). Risk of cerebral vasospasm after subarachnoid hemorrhage reduced by statin therapy: a multivariate analysis of an institutional experience. J Neurosurg.

[44] McGirt MJ, Lynch  JR (2002). Simvastatin increases endothelial nitric oxide synthase and ameliorates cerebral vasospasm resulting from subarachnoid hemorrhage. Stroke.

[45] McGirt MJ, Parra  A (2002). Attenuation of cerebral vasospasm after subarachnoid hemorrhage in mice overexpressing extracellular superoxide dismutase. Stroke.

[46] McGirt MJ, Pradilla  G (2006). Systemic administration of simavastatin after the onset of experimental subarachnoid hemorrhage attenuates cerebral vasospasm. Neurosurgery.

[47] Mori  T, Nagata  K (2001). Intracisternal increase of superoxide anion production in a canine subarachnoid hemorrhage model. Stroke.

[48] Nishihashi  T, Trandafir  CC (2006). Hypersensitivity to hydroxyl radicals in rat basilar artery after subarachnoid hemorrhage. J Pharmacol Sci.

[49] O’Driscoll G, Green  D (1997). Simvastatin, an HMG coenzyme A reductase inhibitor, improves endothelial function within 1 month. Circulation.

[50] Ohyama  H, Hosomi  N (2001). Thrombin inhibition attenuates neurodegeneration and cerebral edema formation following transient forebrain ischemia. Brain Res.

[51] Ostrowski  RP, Colohan  AR (2005). Mechanisms of hyperbaric oxygen induced neuroprotection in a rat model of subarachnoid hemorrhage. J Cereb Blood Flow Metab.

[52] Osuka  K, Watanabe  Y (2009). Modification of endothelial nitric oxide synthase through AMPK after experimental subarachnoid hemorrhage. J Neurotrauma.

[53] Pluta  RM (2005). Delayed cerebral vasospasm and nitric oxide: review, new hypothesis, and proposed treatment. Pharmacol Ther.

[54] Pugh  CW, Ratcliffe  PJ (2003). Regulation of angiogenesis by hypoxia: role of the HIF system. Nat Med.

[55] Rubanyi  GM, Polokoff  MA (1994). Endothelins: molecular biology, biochemistry, pharmacology, physiology, and pathophysiology. Pharmacol Rev.

[56] Santhanam  AV, Smith  LA (2005). Role of endothelial NO synthase phosphorylation in cerebrovascular protective effect of recombinant erythropoietin during subarachnoid hemorrhage induced cerebral vasospasm. Stroke.

[57] Schievink  (1997). Intracranial aneurysms. N Engl J Med.

[58] Schubert  GA, Schilling  L (2008). Clazosentan, an endothelin receptor antagonist, prevents early hypoperfusion during the acute phase of massive experimental subarachnoid hemorrhage: a laser doppler flowmetry study in rats. J Neurosurg.

[59] Semenza  GL (2002). Signal transduction to hypoxia inducible factor 1. Biochem Pharmacol.

[60] Sen  O, Caner  H (2006). The effect of mexiletine on the level of lipid peroxidation and apoptosis of endothelium following experimental subarachnoid hemorrhage. Neurol Res.

[61] Shih  HC, Lin  CL (2006). 17beta estradiol inhibits subarachnoid hemorrhage induced inducible nitric oxide synthase gene expression by interfering with the nuclear factor kappa B transsaction. Stroke.

[62] Shih HC, Lin CL (2008). Upregulation of estrogen receptor alpha and mediation of 17beta estradiol vasoprotective effects via estrogen receptor alpha in basilar arteries in rats after experimental subarachnoid hemorrhage. J Neurosurg.

[63] Sugawara  T, Ayer  R (2008). Simvastatin attenuation of cerebral vasospasm after subarachnoid hemorrhage in rats via increased phosphorylation of Akt and endo thelial nitric oxide synthase. J Neurosci Res.

[64] Sugawara  T, Jadhav  V (2009). Thrombin inhibition by argatroban ameliorate early brain injury and improves neurological outcomes after experimental subarachnoid hemorrhage in rats. Stroke.

[65] Tsurutani  H, Ohkuma  H (2003). Effects of thrombin inhibitor on thrombin related signal transduction and cerebral vasospasm in the rabbit subarachnoid hemorrhage model. Stroke.

[66] Vatter  H, Weidauer  S (2007). Persistence of the nitric oxide dependent vasodilator pathway of cerebral vessels after experimental subarachnoid hemorrhage. Neurosurgery.

[67] Vatter  H, Konczalla  J (2007). Characterization of the endothelin B receptor expression and vasomotor function during experimental cerebral vasospasm. Neurosurgery.

[68] Voldby  B, Enevoldsen  EM (1982). Intracranial pressure changes following aneurysm rupture. Part 1: clinical and angiographic correlations. J Neurosurg.

[69] Wardlaw  JM, White  PM (2000). The detection and management of unruptured intracranial aneurysms. Brain.

[70] Wilkins  RH (1990). Cerebral vasospasm. Crit Rev Neurobiol.

[71] Yamaji  T, Johshita  H (1990). Endothelin family in human plasma and cerebrospinal fluid. J Clin Endocrinol Metab.

[72] Yan  J, Chen  C (2006). 2 methoxyestradiol reduces cerebral vasospasm after 48 hours of experimental subarachnoid hemorrhage in rats. Exp Neurol.

[73] Yanagisawa  M, Kurihara  H (1988). Novel potent vasoconstrictor peptide produced by vascular endothelial cells. Nature.

[74] Zhou  C, Yamaguchi  M (2004). Caspase inhibitors prevent endothelial apoptosis and cerebral vasospasm in dog model of experimental subarachnoid hemorrhage. J Cereb Blood Flow Metab.

[75] Zhou ML, Shi  JX (2007). Potential contribution of nuclear factor kappaB to cerebral vasospasm after experimental subarachnoid hemorrhage in rabbits. J Cereb Blood Flow Metab.

[76] Zimmermann  M, Seifer  V (1998). Endothelin and subarachnoid hemorrhage: an overview. Neurosurgery.

